# Factors controlling the distributions of dissolved organic matter in the East China Sea during summer

**DOI:** 10.1038/s41598-020-68863-w

**Published:** 2020-07-16

**Authors:** Jeonghyun Kim, Tae-Hoon Kim, Sang Rul Park, Hyuk Je Lee, Jang Kyun Kim

**Affiliations:** 1 Marine Environmental Research Center, Korea Institute of Ocean Science and Technology (KIOST), Busan, 49111 Republic of Korea; 2 Department of Oceanography, Faculty of Earth Systems and Environmental Sciences, Chonnam National University, Gwangju, 61186 Republic of Korea; 3 Department of Marine Life Sciences, Jeju National University, Jeju, 63243 Republic of Korea; 4 Department of Biological Sciences, Sangji University, Wonju, 26339 Republic of Korea; 5 Department of Marine Sciences, Incheon National University, Incheon, 22012 Republic of Korea

**Keywords:** Biogeochemistry, Ocean sciences

## Abstract

To determine the distribution of dissolved organic matter (DOM) in the East China Sea (ECS) during the summer, we measured the dissolved organic carbon (DOC) and nitrogen (DON), fluorescent dissolved organic matter (FDOM), and chlorophyll *a* (Chl. *a*) in the upper 100-m layer of this region during July and September 2015. The DOC (r^2^ = 0.72 and 0.78 in July and September, respectively) and DON (r^2^ = 0.43 and 0.33) were significantly correlated with salinity, suggesting that the river is the primary origin of DOM. However, we found that at a DOC “pulse” under a salinity ranging from 24 to 35, the extrapolating DOC values (304 ± 11 μM) were twice higher than those with a salinity of close to 0, as found in a previous study. The excess DOC concentration seemed to be attributed to the microbial metabolism during transport from the estuary based on the good relationships between DOC and marine humic-like FDOM (r^2^ = 0.42 and 0.47), as well as the fluorescence, humification, and biological indexes, but showed no correlation with Chl. *a*. Thus, the results of our study indicate that microbial activities can be a significant factor controlling the distribution of DOM in the ECS during summer.

## Introduction

Dissolved organic matter (DOM) in the oceans is one of the largest reservoirs of carbon and nitrogen on Earth, and its distribution and behaviour play crucial roles not only in biogeochemical processes in the ocean but also in the carbon and nitrogen cycles^1^. Dissolved organic carbon (DOC), the carbon component of DOM, can be preserved for thousands of years in the deep open oceans with relatively lower and uniform concentrations because more than 90% of DOC is recalcitrant to microbial utilization in the water column^2,3^. Thus, this carbon fixed in dissolved organic form could contribute to the sinking of atmospheric CO_2_ in the ocean through the biological pump^4,5^. By contrast, high and significant variations in DOC concentrations have been found in the coastal and marginal oceans owing to intense biological activities and large terrestrial inputs^6^. Dissolved organic nitrogen (DON), the nitrogen component of DOM, including urea, amino acids, nucleic acids, and amino sugars, is a primary reservoir of reactive nitrogen in the oceans. When dissolved inorganic nitrogen (DIN) is depleted, DON is the dominant pool of fixed nitrogen, and can become a direct energy source for marine microorganisms^7^. By the microbial carbon pump, microbial transformation of DOM from labile to recalcitrant state, nitrogen was preferentially removed relative to carbon within the DOM pool, then the recalcitrant DOM shows ultimately the high DOC to DON ratio^8^.

The northwestern Pacific marginal seas, including the East China Sea (ECS) and the southern sea off Korea, are among the largest continental shelves in the world^9^. These seas receive huge amounts of freshwater (0.9 × 10^12^ m^3^ year^−1^) from the Changjiang River^10^ and seawater from the Kuroshio branch water, a strong western boundary current^11^. Here, the mixing of freshwater driven by the Changjiang River with seawater forms a less saline water mass with a salinity of less than 32, which is known as the Changjiang Diluted Water (CDW). The CDW front generally extends northeastward toward the vicinity of Jeju Island, Korea during the summer^12^. The formation of CDW is not simply the result of a water mixing process, but is also influenced by the addition of large amounts of terrestrial materials, triggering a subsequent biogeochemical impact on the adjacent seas. The DIN and DOC fluxes from the Changjiang River to the ECS have been estimated to be 1.5 × 10^12^ g N year^-1^ and 4.8 × 10^12^ g C year^−1^, respectively^13,14^. More than 99% of DIN input from the Changjiang River was removed within 200 km, perhaps due to rapid consumption of DIN by phytoplankton^15^. Especially, in this region, the very enhanced chlorophyll *a* concentration (up to 20 μg L^−1^) was observed over a distance of 100 km from the Changjiang River mouth^16^. Whereas, DON was conservatively mixed (3.4–10.1 μM [avg. 7.0 ± 1.3 μM]) during long-range transport (200–800 km) in the surface layer of the ECS during summer^15^.

A few studies have recently used absorption and fluorescence spectroscopy to characterise DOM in the ECS close to the Changjiang River estuary^17–19^. However, the behaviours of DOC and DON coupled with optical spectroscopy in the ECS and the southern sea off Korea remain unclear owing to a highly dynamic and complex current system^20,21^. Excitation-emission matrix fluorescence coupled with the parallel factor analysis (EEM-PARAFAC) has been applied to evaluate the dynamics of fluorescent groups of DOM (FDOM) in coastal environments^22–24^. Therefore, the objectives of this study were to identify the optical properties of DOM as a tracer of the DOM source and to evaluate the factors controlling the distributions of DOM in the northern ECS and the southern sea off Korea during the summer.

## Results

### Hydrography

The temperature and salinity in the upper 100-m layer ranged respectively from 12.84 °C to 24.43 °C (avg. 18.89 ± 3.91 °C) and 24.29 to 34.80 (avg. 32.53 ± 1.77) in July 2015, and from 11.35 °C to 24.46 °C (avg. 18.24 ± 5.01 °C) and 26.69 to 34.02 (avg. 32.11 ± 1.56) in September 2015, respectively. The highest temperature and lowest salinity were observed in the surface water (0 m) of the southwestern area in July 2015 and the northeastern area in September 2015 (Fig. S1). Here, some water patches with relatively lower salinity (i.e., CDW) showed in the East China Sea and the southern sea of Korea. In the surface water, the temperature was relatively lower in July 2015 (avg. 23.24 ± 0.66 °C) than in September 2015 (avg. 24.12 ± 0.31 °C), while salinity was slightly higher in July 2015 (avg. 30.89 ± 2.01) than in September 2015 (avg. 30.07 ± 1.11).

### Concentration of DIN

The concentration of nitrate (NO_3_^−^) in the upper 100-m layer ranged from 0.25 to 11.7 μM (avg. 3.66 ± 4.04 μM) in July 2015 and from 0.05 to 9.20 μM (avg. 3.78 ± 3.31 μM) in September 2015, respectively. On the other hand, nitrite (NO_2_^−^) was observed to be very depleted: from 0.00 to 0.44 μM (avg. 0.06 ± 0.10 μM) in July 2015 and from 0.00 to 0.25 μM (avg. 0.08 ± 0.06 μM) in September 2015, respectively. The DIN is the sum of nitrate–N and nitrite-N. The concentration of DIN in the upper 100-m layer ranged from 0.28 to 11.7 μM (avg. 3.72 ± 4.06 μM) in July 2015 and from 0.05 to 9.30 μM (avg. 3.86 ± 3.35 μM) in September 2015, respectively, and increased generally with depth. The relatively lower concentration of nutrients was observed in the surface water of north-western areas of the ECS. In the surface layer, the concentration of DIN was relatively higher in July 2015 (avg. 0.44 ± 0.20 μM) than in September 2015 (avg. 0.21 ± 0.21 μM).

### Concentration of DOC and DON

The concentrations of DOC in the upper 100-m layer of the ECS ranged from 60 to 120 μM (avg. 79 ± 14 μM) in July 2015 and from 61 to 113 μM (avg. 84 ± 13 μM) in September 2015. The DOC concentrations in the upper 100-m layer of the ECS were in the upper range and comparable to those of the major world oceans (60–80 μM)^25–27^, but lower than those in the Changjiang estuary, which have been determined by different authors to be approximately 100–170 μM^28,29^. The DON concentrations in the upper 100-m layer of the ECS ranged from 1.89 to 10.6 μM (avg. 5.17 ± 1.76 μM) in July 2015 and from 4.66 to 9.97 μM (avg. 6.90 ± 1.31 μM) in September 2015. The DON concentrations in the ECS were slightly lower than those in the surface layer of the Atlantic Ocean (4–11 µM), Pacific Ocean (7–13 µM)^30–32^, and the Changjiang estuary (14.12 μM)^33^ and its adjacent sea (6.7 μM)^34^, and fell within the range of those of the East/Japan Sea (4–7 μM)^35^. In the surface layer, the concentrations of DOC and DON were relatively lower in July 2015 (avg. 93 ± 10 μM for DOC; 6.25 ± 1.67 μM for DON) than in September 2015 (avg. 100 ± 7 μM for DOC; 7.62 ± 1.01 μM for DON).

### Characterisation and concentration of FDOM

Three fluorescence components were characterised using the PARAFAC model. The peaks of component 1 (C1), component 2 (C2), and component 3 (C3) showed excitation (Ex) and emission (Em) maximum at Ex/Em = 285/334 nm, Ex/Em = 325/402 nm, and Ex/Em = 395(270)/450 nm, respectively (Fig. S2). The three components were matched to components from previous PARAFAC studies according to the OpenFluor database^36^ (Table 1). The spectral properties of the three peaks identified in our study were similar to the protein-like peak T, marine humic-like peak M, and terrestrial humic-like peak C reported by Coble^37^. The intensities of C1, C2, and C3 ranged from 0.49 to 11.59 QSU (avg. 2.98 ± 2.65 QSU), from 0.23 to 1.32 QSU (avg. 0.59 ± 0.23 QSU), and from 0.18 to 2.35 QSU (avg. 0.83 ± 0.42 QSU) in July 2015, and from 0.89 to 7.90 QSU (avg. 2.50 ± 1.35 QSU), from 0.54 to 1.10 QSU (avg. 0.79 ± 0.13 QSU), and from 0.72 to 1.63 QSU (avg. 1.15 ± 0.21 QSU) in September 2015. In the surface layer, the intensity of C1 was relatively higher in July 2015 (avg. 4.65 ± 3.29 QSU) than in September 2015 (avg. 2.95 ± 1.21 QSU), while the intensities of C2 and C3 were relatively lower in July 2015 (avg. 0.71 ± 0.22 QSU for C2; 0.83 ± 0.53 QSU for C3) than in September 2015 (avg. 0.86 ± 0.11 QSU for C2; 1.16 ± 0.17 QSU for C3).

**Table 1:** The spectral information and description of the fluorescent components identified using the parallel factor analysis model in the East China Sea.

Component	Max. wavelength (Ex/Em, unit: nm)	Description	Coble 2007	Number of matches	Previous studies
1	285 / 334	Protein-like, amino acid-like	T	6	C7 (Murphy, et al.^66^)
		Biological production			C5 (Murphy, et al.^67^)
		Freshly production			C5 (Walker, et al.^68^)
2	325 / 402	Marine humic-like	M	3	C2 (Catalá, et al.^69^)
		Microbial activities			C2 (Dalmagro, et al.^70^)
		Ubiquitous Humic-Like			C2 (Liu, et al.^71^)
3	395(270)/450	Terrestrial humic-like	C	5	C2 (Dainard, et al.^72^)
		UV/visible humic-like			C1 (Yamashita, et al.^73^)
		High MW plant-derived material			C3 (Peleato, et al.^74^)

Three components were compared with previous studies using the OpenFluor database^36^.

### Concentration of chlorophyll a

The concentration of chlorophyll *a* (Chl. *a*) in the upper 100-m layer ranged from 0.17 to 3.64 mg m^−3^ (1.00 ± 0.91 mg m^−3^) in July 2015 and from 0.04 to 1.04 mg m^−3^ (0.37 ± 0.27 mg m^−3^) in September 2015, respectively. In the surface layer, the Chl. *a* concentration in July 2015 (avg. 0.6 ± 0.4 mg m^−3^) was similar with September 2015 (avg. 0.5 ± 0.3 mg m^−3^).

## Discussion

**Figure 1:**
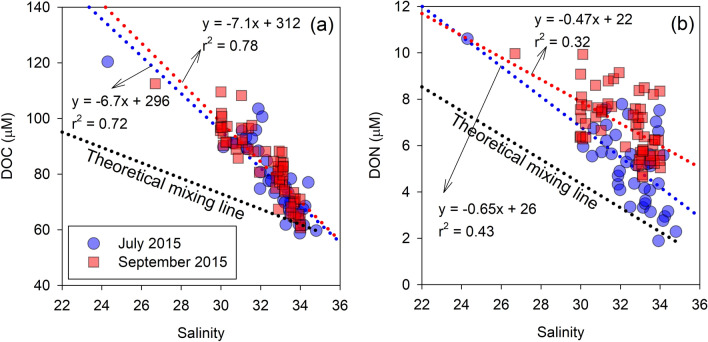
Scatterplots of the DOC (**a**) and DON (**b**) concentrations against salinity in July and September 2015. The blue- and red-dotted lines indicate regression lines for each sampling cruise. The black-dotted lines indicate the theoretical mixing line of the end-members between seawater with the highest salinity and the Changjiang River water derived from previous studies (156 μM for DOC^29^ and 20 μM for DON^42^). The Pearson correlation analysis was conducted using SigmaPlot Version 12.0 (Systat Software, CA) (www.systatsoftware.com).

The relationships of DOC and DON against salinity during the two cruises are shown in Fig. 1. DOC was significantly correlated with salinity in July and September 2015 (r^2^ = 0.72, *p* < 0.001 and r^2^ = 0.78, *p* < 0.001). In addition, DON was weakly correlated with salinity in July and September 2015 (r^2^ = 0.43, *p* < 0.001 and r^2^ = 0.32, *p* < 0.001) compared to DOC. These relationships suggest that the river is the primary origin of DOC and DON. Because the study region is close to Jeju Island and the southern coast of Korea peninsula (Fig. S3), the potential influence of the land-origin DOM from those places could be expected. However, the DOC concentration in the coastal groundwater of Jeju Island, in which the major source of terrestrial materials is submarine groundwater discharge, was reported to be very depleted (26 ± 11 μM^38^ and 21–56 μM^39^). Assuming the input of DOC from the southern coast of Korea was significant, the relationship between DOM and salinity should show a large deviation or two mixing lines due to the effect by the two different endmember. Here, we generally observed one mixing line in this study (Fig. 1). In addition, the Changjiang River, which is one of the world’s largest rivers, accounts for approximately 90% of the freshwater input to the adjacent seas and near Korea peninsula^40,41^. These indicate that the Changjiang River is the primary source of DOM, corresponding the previous study^15^.

However, we found that a DOC “pulse” occurred at a salinity ranging from 24 to 35 (Fig. 1), implying significant discrepancies in the DOC concentration between the extrapolated DOC values of the DOC-salinity relationship and the values with a salinity of close to 0, as described in a previous study^29^. The extrapolated DOC concentration at zero salinity in this study (304 ± 11 μM for both periods) was approximately twice that previously reported in the Changjiang River (156 ± 28 μM)^29^. This indicates that the DOC originating from the Changjiang River was not conservatively mixed in this region. In a previous study, the DOC pulse in the Changjiang estuary and the adjacent ECS shelf occurred within salinity ranges of approximately 19–27 in July 2015^29^. They suggested that the increase in DOC was related to the high percentage of particulate organic carbon (%POC) in suspended particulate matter (SPM) by algal accumulation or bloom based on the SPM size spectra measured using laser *in-situ* scattering and transmissometry.

By contrast, the extrapolated DON concentration at zero salinity (26 ± 3 μM) was similar to the concentration of DON in the Changjiang River waters (approximately 20 μM)^42^. This suggests that the riverine DON was consistent and mixed conservatively in the ECS. Hopkinson, et al.^43^ showed that the average half-lives of labile and semi-labile DON are 12 and 113 days, respectively, based on incubation experiments using the continental shelf water of the middle Atlantic bright. Here, the CDW from the estuary mouth took 33 ± 2 days to arrive within the vicinity of Jeju Island (~ 450 km) using a radioactive decay equation of ^223^Ra activities^44^. The conservative behaviour of the Changjiang River-driven DON in this study corresponds to the results from Kwon, et al.^15^ which showed a conservative mixing pattern of DON of up to 800 km from the Changjiang River mouth.

**Figure 2:**
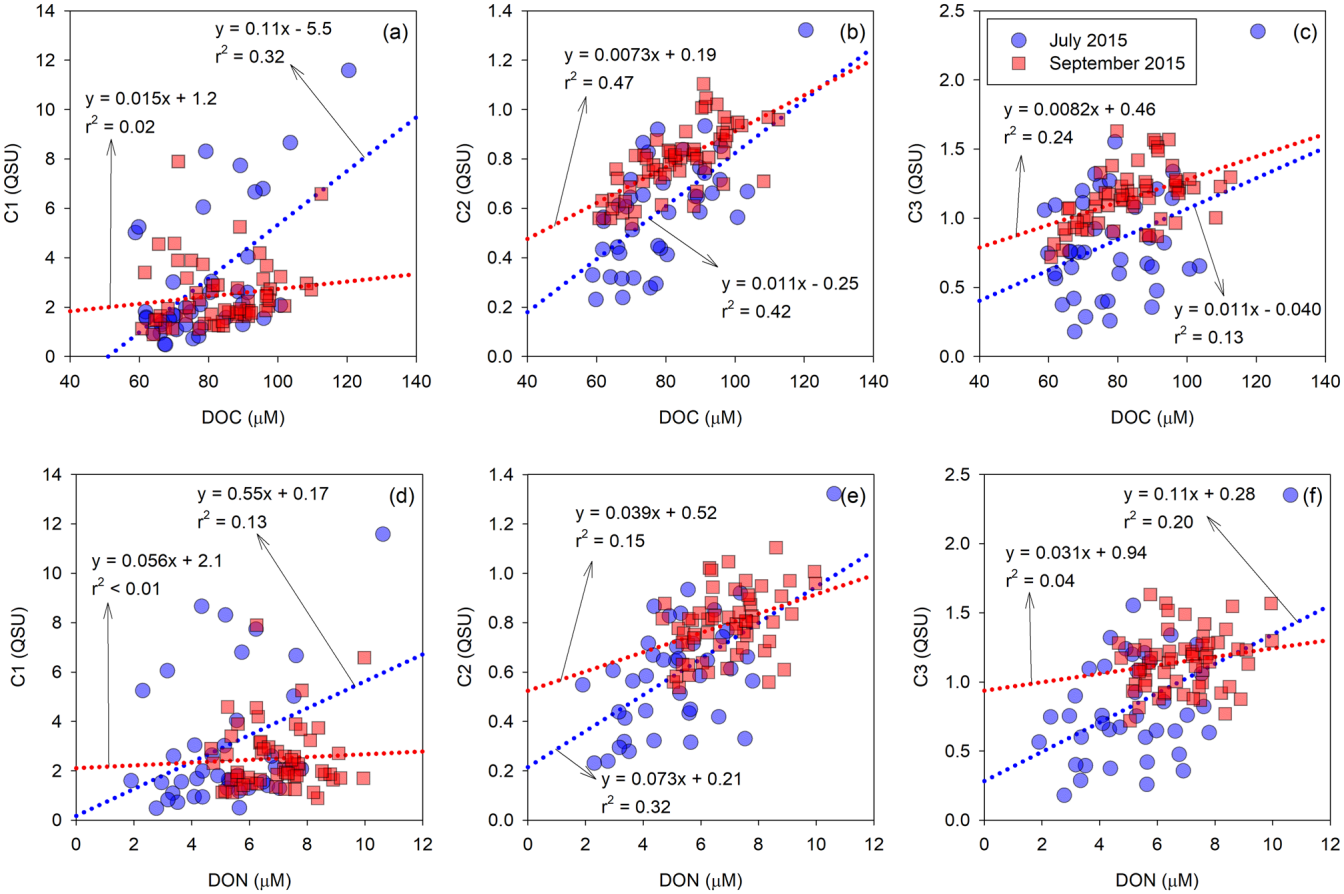
Scatterplots of fluorescent intensities of FDOM components against the DOC (**a**–**c**) and DON (**d**–**f**) concentrations in July and September 2015. The dotted lines indicate regression lines for each sampling cruise. The Pearson correlation analysis was conducted using SigmaPlot Version 12.0 (Systat Software, CA) (www.systatsoftware.com).

**Figure 3:**
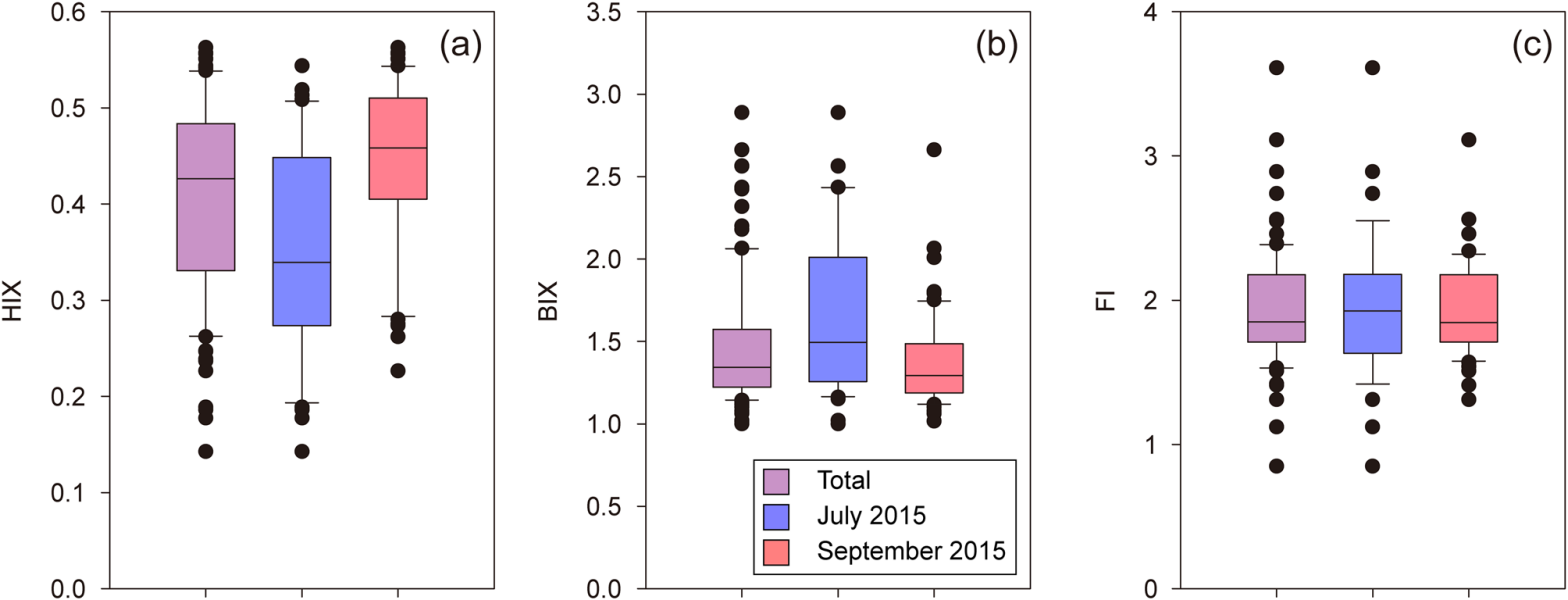
Box plots of HIX (**a**), BIX (**b**), and FI (**c**) for the total time period (purple), July (blue), and September (red) 2015.

To evaluate the source of the DOC pulse in this study, we compared DOC concentrations with the fluorescent properties of DOM (Fig. 2). The relationships between DOC and C2 in the ECS showed significant positive trends in July and September 2015 (r^2^ = 0.42, *p* < 0.001 and r^2^ = 0.47, *p* < 0.001), whereas the relationships of DOC with C1 and C3 were weak and widely scattered (Fig. 2). This demonstrates that marine humic-like FDOM attributed to microbial processing of organic matter can be an important component of the DOC. To support the microbial activity as the origin of the DOC pulse, we calculated the fluorescence index (FI), humification index (HIX), and biological index (BIX) from the EEM spectral data. The FI has been used to identify the source of humic DOM originating from terrestrial (~ 1.4) or microbial activity (~ 1.9) ^45^. In this study, the value of FI ranged from 0.9 to 3.6 (avg. 1.9 ± 0.5) in July 2015 and from 1.3 to 3.1 (avg. 1.9 ± 0.3) in September 2015 (Fig. 3), generally indicating the microbial origin of DOC in the water column. A few lower FI values may be associated with the Changjiang River origins. The values of HIX, an indicator of the degree of DOM humification ^46^, ranged from 0.1 to 0.5 (avg. 0.4 ± 0.1) in July 2015 and from 0.2 to 0.6 (avg. 0.4 ± 0.1) in September 2015 (Fig. 3). Despite large variations, lower values of less than 4 indicated that DOM in the ECS was related to autochthonous DOM rather than the terrestrial origin^47^. The values of BIX, an indicator of autochthonous biological production^48^, ranged from 1.0 to 2.9 (avg. 1.7 ± 0.5) in July 2015 and from 1.0 to 2.7 (avg. 1.4 ± 0.3) in September 2015 (Fig. 3). The average BIX values were higher than 1.0, which correspond to the freshly produced DOM^48^, and are similar to the values observed in marine surface oligotrophic waters^49^.

Although the photodegradation could modify the FDOM indexes during the long-range transport from the Changjiang River, such as significant decreasing the degree of DOM humification and increasing the BIX value of DOM^50–53^, the values of the indexes between the surface (0 m) and subsurface water (> 0 m) depths in these well-stratified water columns showed no statistically differences (Student’s t-test; p-value = 0.06 for HIX, 0.47 for BIX, and 0.35 for FI). In the previous studies, the relatively lower HIX values (< 2) and higher BIX values (> 0.8) already reported near the Changjiang River mouth^54,55^, indicating that the in-situ biological production seems to be a significant origin of DOM in the ECS. Therefore, the FDOM indexes can be proper indicators to suggest the origin of DOM in the Changjiang River water-seawater mixing zone.

**Figure 4:**
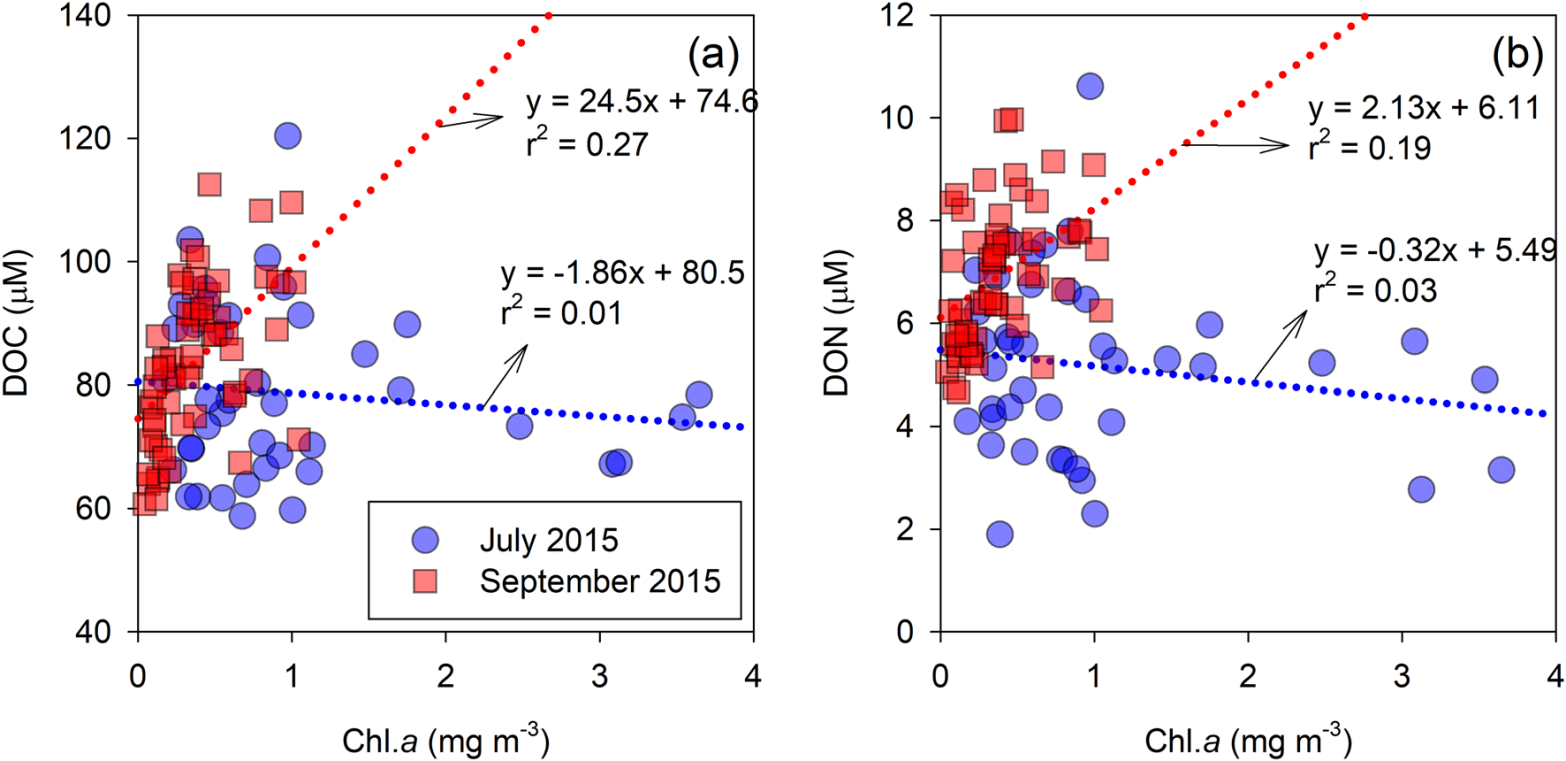
Scatterplots of the DOC (**a**) and DON (**b**) concentrations against the chlorophyll *a* concentration in July and September 2015. The dotted lines indicate regression lines for each sampling cruise. The Pearson correlation analysis was conducted using SigmaPlot Version 12.0 (Systat Software, CA) (www.systatsoftware.com).

In general, the production of autochthonous DOC from phytoplankton biomass in the ocean is considered to be an important source. The highest value of primary production (> 2,000 mg C m^−2^ day^−1^) has been observed in the inner shelf of the ECS during summer, displaying three-times higher values than during other seasons^56^. However, in this study, the depth-averaged concentration of Chl. *a*, an indicator of phytoplankton biomass^57^, within the upper 100-m depth was relatively lower than those (2–5 mg m^−3^) reported for the inner shelf of the ECS during summer^58^. In this study, weak relationships were shown between Chl. *a* and DOC (Fig. 4). However, it was reported that %POC was positively correlated with Chl. *a* in the Changjiang estuary and adjacent ECS shelf in July 2015^29^. In addition, the bacterial biomass (BB) and production (BP) were high in shelf waters of the ECS (no deeper than 50 m) during summer, and the BB increased with the concentrations of Chl. *a* within the range of 0.02–3.00 mg m^−3^, and temperature within the range of 8–20 °C, derived from generalised additive models^59^. Thus, our results indicate that the DOC pulse observed seems to be associated with microbial activities in the seawater rather than the terrestrial and/or phytoplankton-derived DOC.

**Figure 5:**
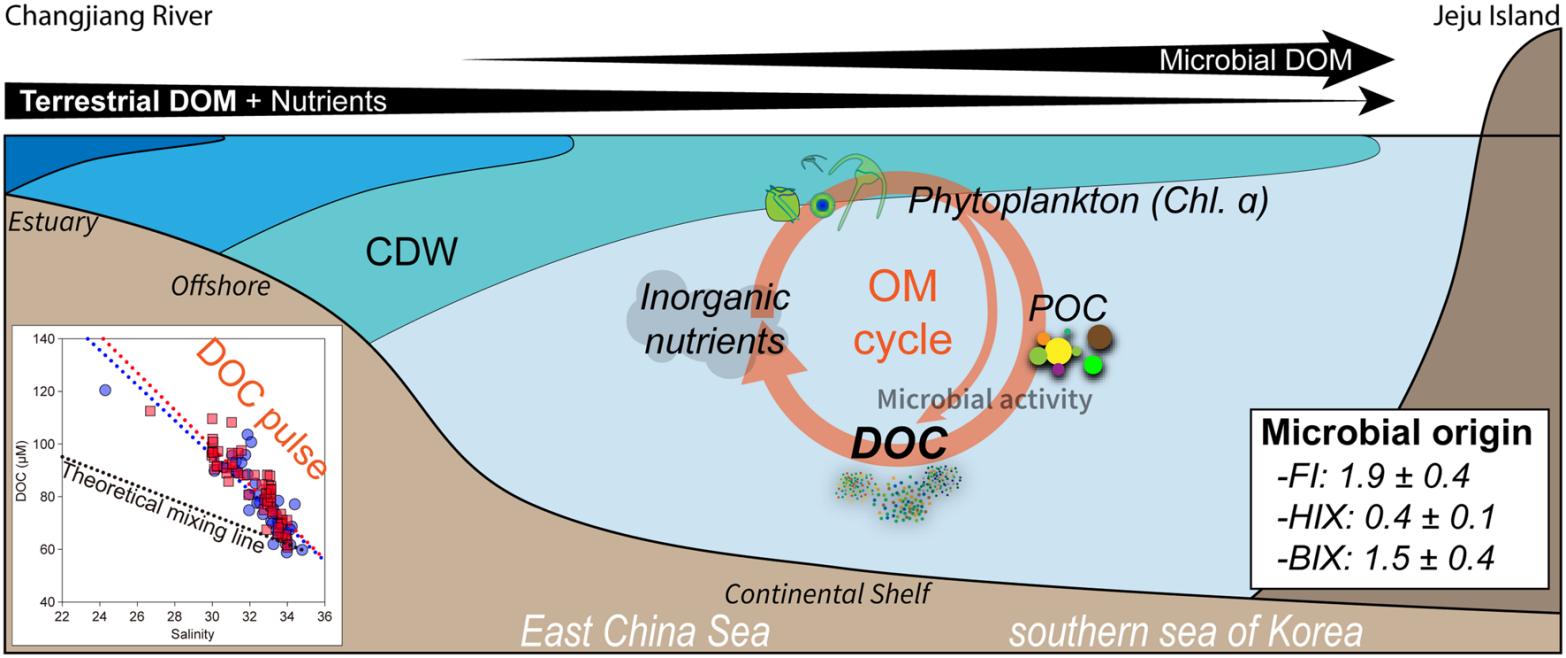
A schematic diagram showing the dynamics of the “DOC pulse” in the East China Sea. The black arrows indicate the transport and addition of the DOM and nutrients from the allochthonous and autochthonous origins, and its thickness means the contribution to the DOM budget in the samples. The average and standard deviation values of FI, HIX, and BIX were calculated from both sampling periods. This figure was drawn using Adobe Illustrator Version 24.1.1. (www.adobe.com).

The behaviours of DOM in this region was elucidated using a schematic diagram of DOC and the optical properties of DOM (Fig. 5). The significant correlations of DOC and DON against salinity and large variations of the FDOM indexes indicated that the DOM in this region seems to originate from the river and its contribution (i.e., allochthonous source) to the DOM budget is likely to be conservatively diminished during the long-range transport from the river mouth. In addition, the inorganic nutrients and organic matter originating from the Changjiang River may fuel the phytoplankton blooming, followed by POM is produced and released by the phytoplankton and zooplankton. The POM would be transformed into DOM by the heterotrophic microbial activity (cell lysis and extracellular release), and successively into inorganic nutrients. Based on the optical properties of DOM, the in-situ production of DOM by the bacteria and virus (i.e., autochthonous source) results in the DOC pulse and the enrichment of the marine humic-like FDOM in the CDW region. According to the result of a previous study using the incubation experiment, phytoplankton-derived organic matter is associated with the formation of the humic-like FDOM^60^. This result supports our arguments. Here, the marine humic-like FDOM and additionally produced DOC in the CDW seems to be simultaneously transported through the ECS. Thus, during the transport of CDW, the contribution of the allochthonous DOM should be decreased, while the autochthonous DOM is likely responsible for the larger fraction of the DOC budget far from the coastal region.

## Conclusions

The distributions of DOC and DON, together with FDOM and Chl. *a*, were measured in the upper 100-m layer of the ECS during summer. In this study, the DOC and DON concentrations were found to be relatively lower than those in the Changjiang estuary. The significant negative correlation between DOM and salinity suggests that the river is the primary source of DOM in the ECS. The expected DOC values extrapolated from the DOC-salinity relationship are twice higher than the DOC measurements in the Changjiang River freshwater. However, the extrapolated DON concentrations were similar to the DON end-member with a salinity of close to 0. The excess DOC relative to the theoretical mixing line implies that the Changjiang River seems to not be the major source of DOC over the ECS during the summer. According to the positive relationship between DOC and the microbial-origin FDOM component, the values of FI, HIX, and BIX, and the relationship between DOC and Chl. *a*, i.e., the main source of the DOC pulse in the ECS, seem to be associated with microbial DOM. Extensive studies on biogeochemical parameters, such as amino acids, δ^13^C, and δ^14^C, will be necessary to understand the biogeochemical behaviour of DOM in highly dynamic and active oceans.

## Materials and methods

### Sampling and hydrological measurements

The hydrological and biogeochemical surveys were conducted during two different periods: July 18 to 20, 2015 aboard the R/V *A-Ra* of Jeju National University (JNU), Korea, and September 21 to 23, 2015 aboard the R/V *Je-Ra* of JNU (Fig. S3). Seawater samples for the vertical profiles were collected from three to six different depths using 12-L Niskin bottles attached to a CTD rosette system. We took the water samples at three different water depths (0, 30, 50 m) during July 2015 and at six depths (0, 20, 40, 60, 80, 100 m) during September 2015, respectively. The temperature, salinity, and Chl. *a* were measured using an SBE911-plus CTD profiler with an ECO-FLD fluorometer (Seabird Electronics, USA). To correct the CTD fluorescence as the Chl. *a* signal, 500 ml water samples were vacuum filtered through Whatman GF/F filters (pore size of 0.7 μm), and then the filters were flash-frozen in liquid nitrogen. The filters were placed in 100% acetone for 4 h to extract the pigment. The Chl. *a* concentration in extracts was determined using a fluorometer (10-AU-005-CE, Turner Designs, CA) just after each sampling cruise.

### Analysis of DOC and DON

Samples for DOC and DON were vacuum filtered using pre-combusted (500 °C for 5 h) Whatman GF/F filters. To prevent microbial degradation, the filtrate was acidified to pH 2 using 6 M HCl, followed by hermetic sealing in pre-combusted ampoules (Wheaton Scientific, Millville, NJ). The concentration of DOC was measured by high-temperature catalytic oxidation (HTCO) using a TOC analyser (TOC-V_CPH_, Shimadzu, Japan). Before starting the analysis, the system blank was reduced until the signal from the carbon-free distilled water was stable below the detection limit (< 5 μM). The accuracy of the DOC concentration was checked for each sample batch using deep-sea reference (DSR; 41–44 μM, University of Miami) samples. The results of our DSR measurement were in good agreement with the consensus values (within 2%). The DON concentration was calculated by subtracting the DIN from the total dissolved nitrogen (TDN), which was measured simultaneously with DOC using the same TOC analyser equipped with a total nitrogen (TN) unit. The concentration of DON was also verified with a precision of 2%–3% of the DSR values (31.00–33.00 μM).

### Analysis of FDOM

Samples for FDOM were filtered simultaneously using GF/F filters along with DOC and DON samples. The filtrate was stored in pre-combusted dark EPA glass vials (Fisher Scientific, PA) to avoid photo-degradation and kept in a refrigerator at below 4 °C until further analysis. Three-dimensional fluorescence spectroscopy was applied using a spectrofluorometer (FS-2, SCINCO, Korea) within 3 days of filtration. The excitation–emission matrix (EEM) fluorescence was measured using scanning emission fluorescence over a wavelength of 250–600 nm at 2-nm intervals with an excitation wavelength of 250–500 nm at 5-nm intervals. The fluorescence data were normalised every day to those of a quinine sulphate dihydrate standard (unit: QSU). Rayleigh and Raman scattering peaks were removed and replaced with a three-dimensional Delaunay interpolation of the remaining data^61^. Parallel factor analysis (PARAFAC) modelling for the compilation of 99 EEM data from the two sampling cruises was conducted using the MATLAB R2019b program with the DOMFluor toolbox^62^. The precision of the FDOM measurement was ± 0.01 QSU, and the detection limit was 0.14 QSU^63^. Fluorescence intensities are reported in quinine sulfate units (QSU). The inner filter effect was not corrected for because the influence of this artifact was negligible using this spectrofluorometer^11,38^.

The FI was calculated as the ratio of the emission fluorescence intensities measured at wavelengths 470 and 520 nm with an excitation wavelength of 370 nm ^64^. The HIX was calculated as the ratio between the sum of the emission fluorescence spectra over the range of 435–480 nm and over the range of 300–345 nm and 435–480 nm at an excitation wavelength of 255 nm ^65^. The BIX was calculated as the ratio between the fluorescence intensity at the emission wavelength of 380 nm to that at 430 nm, at an excitation wavelength of 310 nm^48^.

### Analysis of DIN

Samples for DIN were filtered simultaneously with the DOM samples. The filtrate was transferred to acid-clean polypropylene Nalgene bottles and stored in a freezer (− 20 °C) before analysis. Nitrate and nitrite concentrations were photometrically measured from duplicate samples using a nutrient auto-analyser (QuAAtro39, SEAL Analytical, UK). The accuracies of the nitrate and nitrite concentrations were validated for each sample batch using two different certified reference materials (CRMs): RMNS (31.00 ± 0.24 μM; KANSO, Japan) and MOOS-3 (26.6 ± 0.3 μM; National Research Council of Canada). The results of the CRM measurement were in good agreement with the certified values (within 5%). The detection limits of the DIN components were 0.01 μM.

### Data treatment and statistical analysis

To explore the relationships between the variables, Pearson correlation analysis was conducted using SigmaPlot Version 12.0 (Systat Software, Inc., CA) (www.systatsoftware.com). The reported values of the measurement were shown as the average and standard deviation values using Microsoft Excel 2016 (Microsoft, WA).

## Supplementary information

Supplementary Information.
