# Supplementary material for: Factors controlling the distributions of dissolved organic matter in the East China Sea during summer
Source: Sci Rep. 2020 Jul 16;10:11854. doi: 10.1038/s41598-020-68863-w (PMC7366708; doi:10.1038/s41598-020-68863-w)
Supplement: Supplementary file 1 — Supplementary Information. [file 41598_2020_68863_MOESM1_ESM.docx]

*Scientific Reports*

Supporting Information for

**Factors controlling the distributions of dissolved organic matter in the East China Sea during summer**

*Jeonghyun Kim^1^*, *Tae-Hoon Kim*^2^, *Sang Rul Park*^3^, *Hyuk Je Lee*^4^, *Jang Kyun Kim*^5^

^1^Marine Environmental Research Center, Korea Institute of Ocean Science and Technology (KIOST), Busan 49111, Republic of Korea

^2^Department of Oceanography, Faculty of Earth Systems and Environmental Sciences, Chonnam National University, Gwangju 61186, Republic of Korea

^3^Department of Marine Life Sciences, Jeju National University, Jeju 63243, Republic of Korea

^4^Department of Biological Sciences, Sangji University, Wonju 26339, Republic of Korea

^5^Department of Marine Sciences, Incheon National University 22012, Republic of Korea

**Contents of this file:** Figures S1, S2, and S3

This Supporting Information contains three supplementary figures referred to in the article text.

**
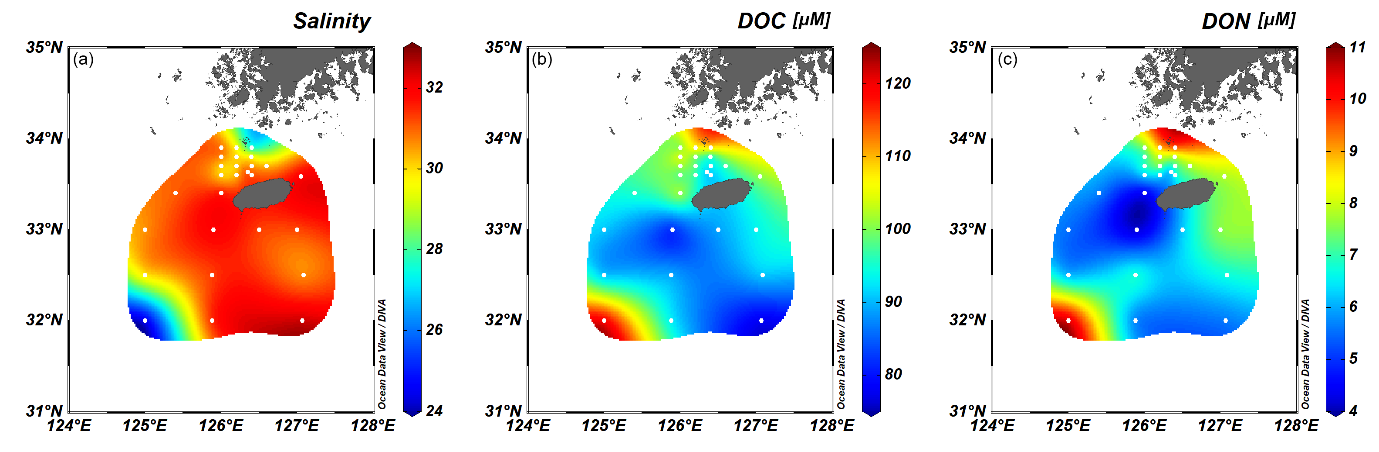
**

**Figure S1.** Contour figures of salinity (a), DOC (b), and DON (c) in surface waters of the East China Sea and the southern sea of Korea in the July and September 2015.

**
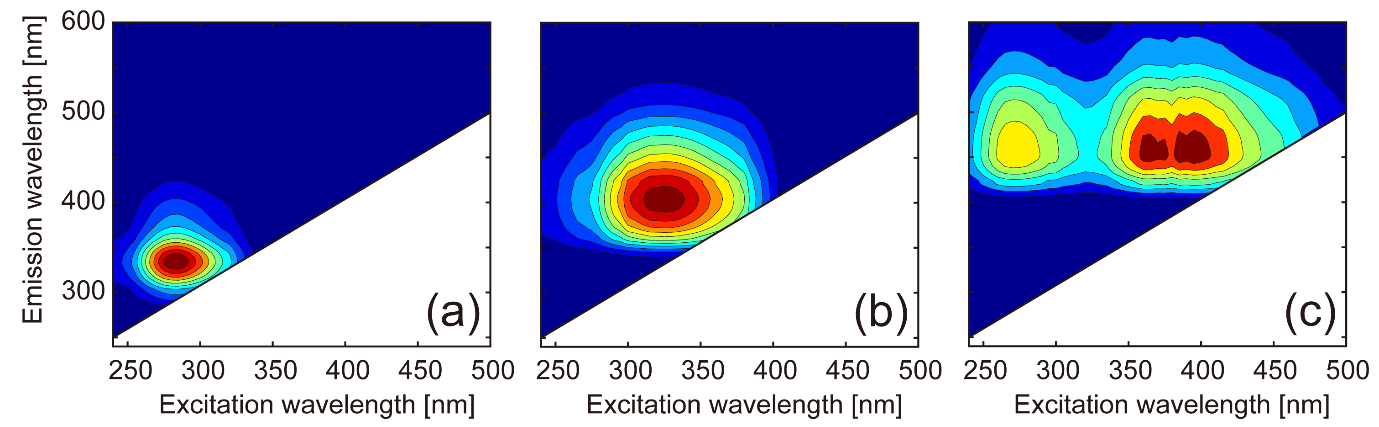
**

**Figure S2.** Contour plots of EEMs for the three components identified using the PARAFAC model.

**
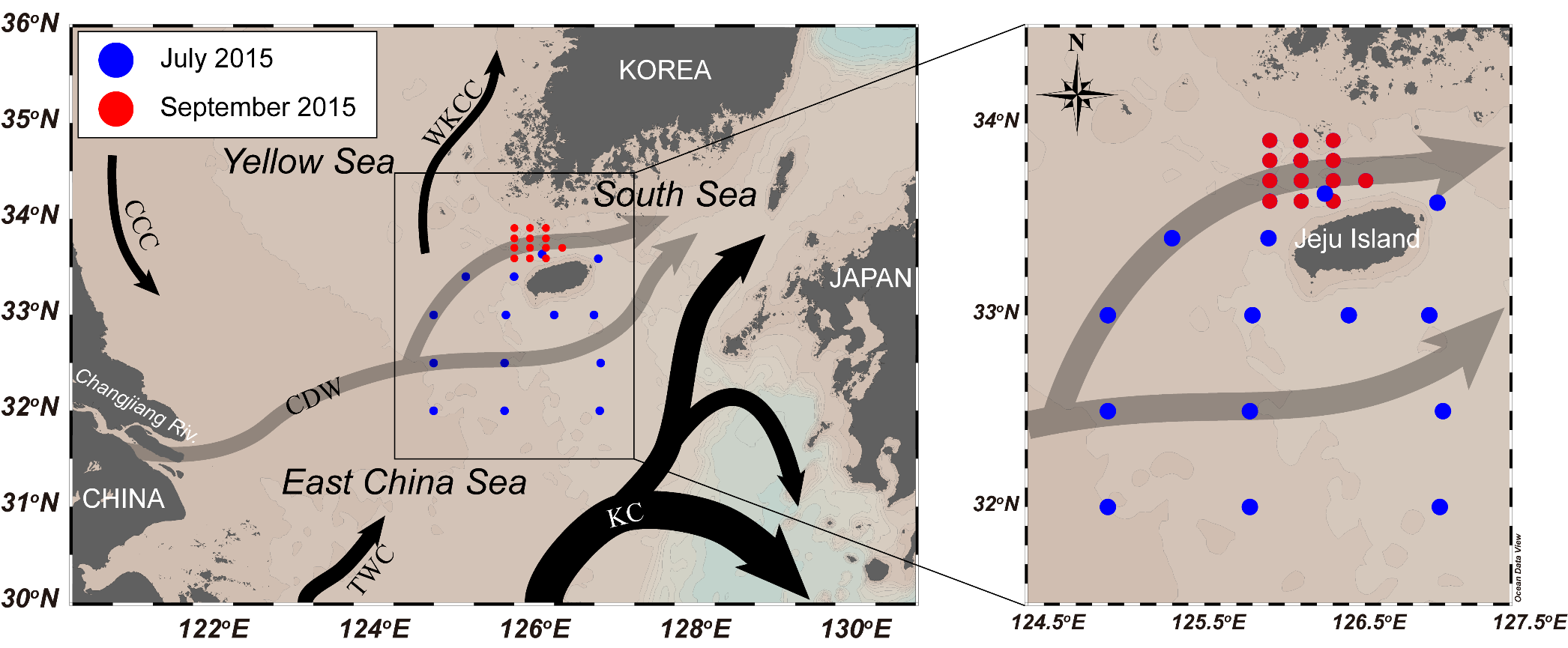
**

**Figure S3.** Maps showing sampling stations in July (blue) and September 2015 (red), and the surface currents and bottom topography of the northwestern Pacific Ocean, including the East China Sea and the southern sea off South Korea. The black arrows represent the seawater currents, and the semi-transparent arrows represent the Changjiang Diluted Water (CDW) current in the summer. KC, TWC, WKCC, and CCC denote the Kuroshio Current, Taiwan Warm Current, West Korea Coastal Current, and China Coastal Current, respectively. The maps were created using Ocean Data View, version 5.2.0. (https://odv.awi.de), and the sampling stations and current patterns were drawn using Adobe Illustrator, version 24.1.1. (<http://www.adobe.com/>).
